# Cost-effectiveness analysis of sintilimab additional to chemoradiotherapy in high-risk locoregionally advanced nasopharyngeal carcinoma

**DOI:** 10.3389/fphar.2025.1548710

**Published:** 2025-07-09

**Authors:** Longjiang She, Siqi Tang, Jiaqi Han, Guichao Liu, Lusi Chen, Yang Zhang, Weijun Luo, Weihan Zuo, Feng Ma, Yan Xiong, Ning Zhang

**Affiliations:** ^1^ Department of Nasopharyngeal Oncology, First People’s Hospital of Foshan, Foshan, China; ^2^ Foshan Key Laboratory of Precision Therapy in Oncology and Neurology, First People’s Hospital of Foshan, Foshan, China; ^3^ Department of Head and Neck Oncology, Cancer Center and State Key Laboratory of Biotherapy, West China Hospital, Sichuan University, Chengdu, China; ^4^ Department of Radiation Oncology, Cancer Center and State Key Laboratory of Biotherapy, West China Hospital, Sichuan University, Chengdu, China

**Keywords:** sintilimab, immunotherapy, cost-effectiveness analysis, high-risk locoregionally advanced nasopharyngeal carcinoma, chemoradiotherapy

## Abstract

**Objectives:**

The recently released CONTINUUM trial was the first phase 3 randomized study to demonstrate the efficacy and safety of immunotherapy in high-risk locoregionally advanced nasopharyngeal carcinoma (NPC), showing that sintilimab can bring clinical benefits to these populations.

**Materials and Methods:**

We developed a Markov model to assess the cost and effectiveness of sintilimab plus standard therapy versus standard therapy alone. The primary outcomes included total costs, life-years, quality adjusted life years (QALYs) and incremental cost-effective ratios (ICERs). A series of sensitivity analyses were conducted to test the stability of the model.

**Results:**

When compared to standard therapy, the addition of sintilimab yielded extra 3.10 QALYs at an increased cost of $24208.60, resulting in an ICER of $7819.669 per QALY. Our one-way sensitivity analysis indicated that the utility of event-free survival and the risk of leukopenia/neutropenia in immunotherapy group were the most influential factors impacting the results. The incorporation of sintilimab alongside standard therapy demonstrated a 95.4% probability of being cost-effective.

**Conclusion:**

First-line induction-concurrent chemoradiotherapy with sintilimab was identified as a cost-effectiveness treatment option for high-risk locoregionally advanced NPC.

## Introduction

Nasopharyngeal carcinoma (NPC), a head and neck malignancy linked to Epstein-Barr virus (EBV) infection (Chen Y-P. et al., 2019), demonstrates distinctive epidemiological patterns with endemic prevalence in South China and Southeastern Asia (Sung et al., 2021). Approximately 70% of patients present with locoregionally advanced disease at initial diagnosis (Yang et al., 2019). Current guidelines recommend induction chemotherapy in combined with concurrent chemoradiotherapy as first-line treatment for this population (Bossi et al., 2021; Caudell et al., 2022). Despite this intensive regimen, 20%–30% of high-risk locoregionally advanced NPC patients (stage III-IVa excluding T3-4N0 and T3N1, American Joint Committee on Cancer 8th edition TNM staging) develop locoregional relapse or distant metastasis (Zhang et al., 2019; Sun et al., 2016; Liu et al., 2024; Chen et al., 2021). This unmet medical need underscores the imperative for the novel therapeutic strategies targeting this subgroup. Since the 21st century, immune checkpoint inhibitors (ICIs) has garnered increasing attention. The latent membrane protein 1 induced by EBV has been found to upregulate the expression of programmed cell death protein 1 ligand (PD-L1) (Zhang et al., 2015; Fang et al., 2014). The notable characteristic of NPC is the elevated expression of PD-L1 (ranging from 83%–92%) and significant infiltration of non-malignant lymphocytes, indicating the potential efficacy of immunotherapy in treating this type of cancer (Mai et al., 2023; Larbcharoe et al., 2018).

A recent phase 3 randomized controlled trial, known as CONTINUUM, has demonstrated the survival benefit of sintilimab, a programmed cell death protein (PD-1) inhibitor, combined with chemoradiotherapy in the first-line treatment for patients with locoregionally advanced NPC(8). In this trial, the group receiving sintilimab exhibited a significant improvement in event-free survival (EFS) compared to the standard therapy group (36-month rate, 86% vs. 76%; hazard ratio, 0.59; p = 0.019), albeit with higher but manageable adverse events (AEs). Consequently, based on these compelling findings, the CONTINUUM study has been selected as a Late-breaking Abstract by American Society of Clinical Oncology (ASCO) in 2023 and this first-line treatment protocol is expected to be included in the 2024 Chinese Society of Clinical Oncology (CSCO) clinical guidelines.

Although this treatment regimen has brought clinical benefits, addition of immunotherapy undoubtedly places a greater economic burden on patients and healthcare systems. Therefore, our study aimed to appraise the potential economic implications associated with adding sintilimab to standard chemoradiotherapy as first-line treatments for patients with locoregionally advanced NPC from the Chinese citizen’s perspective.

## Materials and methods

### Model structure

We utilized TreeAge Pro 2019 (TreeAge Software Inc., Williamstown, MA) and employed a Markov model to conduct a comprehensive cost-effectiveness analysis. The Markov cycle length was set as 3 weeks to align with the treatment cycle. A 5% annual discount rate was applied to cost and effect from the perspective of the Chinese payer (Wu et al., 2018). The primary outcomes measured in this analysis included total costs, life years (LYs), quality-adjusted life years (QALYs), and incremental cost-effectiveness ratios (ICERs). The development process was simulated using three states: EFS, progression disease (PD), and death (Supplementary Figure S1). Following disease progression, the participants could receive subsequent therapies. All analyses were based on data derived from the CONTINUUM trial.

### Model survival and transitions estimates

We estimated the transition probabilities between survival states by analyzing the EFS and overall survival (OS) curves from the CONTINUUM trial. To extract the data points from the OS Kaplan–Meier curves, we employed GetData Graph Digitizer (version 2.25; http://www.getdata-graph-digitizer.com/index.php) (Hoy et al., 2011). The process involved: calibrating axes by defining coordinate systems using published scale values; systematically extracting datapoints at predetermined intervals; datapoints underwent duplicate digitization, and averaged values were utilized for subsequent analysis. Next, these data points were fitted to several commonly used parametric survival models, namely, the Log-logistic, Exponential, Weibull, Lognormal, and Gompertz distributions (Latimer, 2013). The selection criterion for choosing the most appropriate model was based on the lowest Akaike’s information criterion (AIC) and Bayesian information criterion (BIC) values. Among these models, the Log-logistic distribution yielded the best fit with the lowest AIC and BIC values compared to the other four methods (Supplementary Table S1). To obtain the shape parameter (γ) and scale parameter (λ), we used the R package (http://www.r-project.org/) based on this optimal fit.

### Utility estimates

Within the context of the CONTINUUM trial, health-related quality of life (HRQOL) was assessed using the European Organisation for Research and Treatment of Cancer (EORTC) Core Quality of Life Questionnaire (QLQ-C30; version 3.0) (Liu et al., 2024). Notably, the HRQOL values were found to be comparable between the sintilimab and standard therapy groups, suggesting similar utility values for both populations. Referring to previous reports, utilities of 0.76 and 0.57 were assigned to the EFS and PD states, respectively, in this analysis (Yang et al., 2020; She et al., 2022).

### Cost inputs

The model incorporated various direct medical costs, such as drugs, radiotherapy, hospitalization, laboratory tests, imaging examinations, AEs management, and subsequent therapy. In the trial, patients were randomly assigned to one of two treatment groups: receiving gemcitabine and cisplatin induction chemotherapy followed by concurrent cisplatin radiotherapy (standard therapy group) or standard therapy combined with sintilimab for 12 cycles (sintilimab group). Disease recurrence patterns and subsequent therapy after progression were considered based on the findings reported in the trial (Liu et al., 2024).

We employed a standardized body surface area of 1.72 m^2^, derived from analogous research findings in China (Chen X. et al., 2019). Grade ≥3 AEs with a frequency of 3% or higher were included in our analysis. The costs associated with AEs were calculated by multiplying the incidence of AEs by the management costs per event. All costs were assumed from previous studies and real-world expenses incurred at the First People’s Hospital of Foshan (Wu et al., 2018; She et al., 2022; Shi et al., 2018; Wu et al., 2012). Conversion of all costs was carried out using an exchange rate of 1 USD = 7.1088 CHY as of June 2024 (22). Detailed information regarding these aspects can be found in Tables 1 and 2.

**TABLE 1:** Key data in CONTINUUM.

Variable	Baseline value (range)	References	Distribution
EFS survival model			
Sintilimab group	Shape = 0.7947444, Scale = 0.0081186	Liu et al. (2024)	-
Standard therapy group	Shape = 1.2297630, Scale = 0.0025608	Liu et al. (2024)	-
OS survival model			
Sintilimab group	Shape = 1.1514328, Scale = 0.0011003	Liu et al. (2024)	-
Standard therapy group	Shape = 2.214, Scale = 0.00001507	Liu et al. (2024)	-
Risk for main adverse events in Sintilimab group			
Anaemia	0.16	Liu et al. (2024)	Beta
Leukopenia	0.25	Liu et al. (2024)	Beta
Nausea	0.14	Liu et al. (2024)	Beta
Mucositis	0.33	Liu et al. (2024)	Beta
Anorexia	0.10	Liu et al. (2024)	Beta
Neutropenia	0.21	Liu et al. (2024)	Beta
Dermatitis	0.04	Liu et al. (2024)	Beta
Fatigue	0.07	Liu et al. (2024)	Beta
Vomiting	0.11	Liu et al. (2024)	Beta
Risk for main adverse events in standard therapy group			
Anaemia	0.11	Liu et al. (2024)	Beta
Leukopenia	0.22	Liu et al. (2024)	Beta
Nausea	0.15	Liu et al. (2024)	Beta
Mucositis	0.30	Liu et al. (2024)	Beta
Anorexia	0.07	Liu et al. (2024)	Beta
Neutropenia	0.18	Liu et al. (2024)	Beta
Dermatitis	0.03	Liu et al. (2024)	Beta
Vomiting	0.11	Liu et al. (2024)	Beta

Abbreviation: EFS, event-free survival; OS, overall survival.

**TABLE 2:** Cost inputs and utilities.

Variable	Baseline value (range)	References	Distribution (parameters)
Body surface area, m^2^	1.72	Chen X. et al. (2019)	
Drug cost, $/cycle			
Sintilimab	303.85 (243.08–364.62)	Local charge	Gamma
Gemcitabine	9.63 (7.7–11.56)	Local charge	Gamma
Cisplatin	11.61 (9.29–13.93)	Local charge	Gamma
Concurrent cisplatin	15.84 (12.38–18.57)	Local charge	Gamma
Antiemetic drugs	65.55 (52.44–78.66)	Local charge	Gamma
Radiotherapy	9,633.84 (7,707.07–11560.61)	Local charge	Gamma
Preparation of radiotherapy	681.52 (545.22–817.83)	Local charge	Gamma
Main subsequent therapy, $/cycle			
Chemotherapy	35.29 (28.23–42.35)	Local charge	Gamma
Immunotherapy	417.47 (333.98–500.96)	Local charge	Gamma
Surgery	4,635.85 (3,708.68–5,563.02)	Local charge	Gamma
2nd radiotherapy	5,081.44 (4,065.15–6,097.73)	Local charge	Gamma
Targeted therapy	558.32 (446.66–669.98)	Local charge	Gamma
Supported care	52.5 (42–63)	Local charge	Gamma
Subsequent therapy in Sintilimab group	300.02 (240.02–360.02)	Local charge	Gamma
Subsequent therapy in standard therapy group	540.6 (432.48–648.72)	Local charge	Gamma
Expenditures on main adverse events, $			
Anaemia	508.2 (406.56–609.84)	Wu et al. (2018)	Gamma
Leukopenia	406.37 (325.10–487.64)	Shi et al. (2018)	Gamma
Nausea	44.3 (35.44–53.16)	Wu et al. (2012)	Gamma
Mucositis	19.94 (15.95–23.93)	Local charge	Gamma
Anorexia	26 (20.8–31.2)	Local charge	Gamma
Neutropenia	406.37 (325.10–487.64)	Local charge	Gamma
Dermatitis	16.64 (13.31–19.97)	Local charge	Gamma
Fatigue	110.3 (88.24–132.36)	Wu et al. (2018)	Gamma
Vomiting	44.3 (35.44–53.16)	Wu et al. (2012)	Gamma
Rash	59.57 (47.66–71.48)	Local charge	Gamma
Hospitalization $/per cycle	126.78 (101.43–152.14)	Local charge	Gamma
Laboratory $/per cycle	74.85 (59.88–89.82)	Local charge	Gamma
Follow-up test $/per time	378.12 (302.5–453.74)	Local charge	Gamma
PICC per time	134.15 (107.32–160.98)	Local charge	Gamma
Discount rate	0.05	Wu et al. (2018)	-
Utility			-
Utility EFS	0.76 (0.61–0.91)	She et al. (2022)	Beta
Utility PD	0.57 (0.46–0.68)	She et al. (2022)	Beta
Death	0	She et al. (2022)	Beta

Abbreviation: EFS, Event-free survival; PD, progressive disease; PICC, peripherally inserted central catheters.

### Sensitivity analysis

To ensure the reliability of our model, we conducted one-way and probabilistic sensitivity analyses. The one-way sensitivity analysis was performed to assess the impact of the parameters on incremental cost-effectiveness ratios (ICERs). On the other hand, the probabilistic sensitivity analysis involved 1,000 resampling iterations to evaluate input parameters and calculate the probability of the sintilimab group being cost-effective compared to the standard therapy group. Furthermore, in subgroup analysis, we evaluated the PD-L1 combined positive score (CPS) and tumor proportion score (TPS) and EBV DNA levels as additional factors influencing the outcomes.

## Result

### Base case results

In comparison to the standard therapy, the addition of sintilimab resulted in an additional 4.24 LYs and 3.10 QALYs, while increased the total cost by $24208.60. Consequently, the ICER for the sintilimab group, as compared to the standard therapy group, was calculated as $5,721.08 per LY and $7,819.67 per QALY, as presented in Table 3.

**TABLE 3:** Baseline results.

Strategies and scenarios	Total cost, $	LYs	QALYs	ICER per LY	ICER per QALY
Sintilimab group	100,097.40	12.51	8.81	5,721.08	7,819.66
EFS state	71,396.07	8.84	6.72	-	-
PD state	28,701.33	3.67	2.09	-	-
Standard therapy group	75,888.80	8.27	5.71	-	-
EFS state	40,446.13	5.24	3.98	-	-
PD state	35,442.67	3.03	1.73	-	-

Abbreviation: ICER, incremental cost-effectiveness ratio; LY, life-year; QALY, quality-adjusted life-year.

### Sensitivity analysis

**FIGURE 1:**
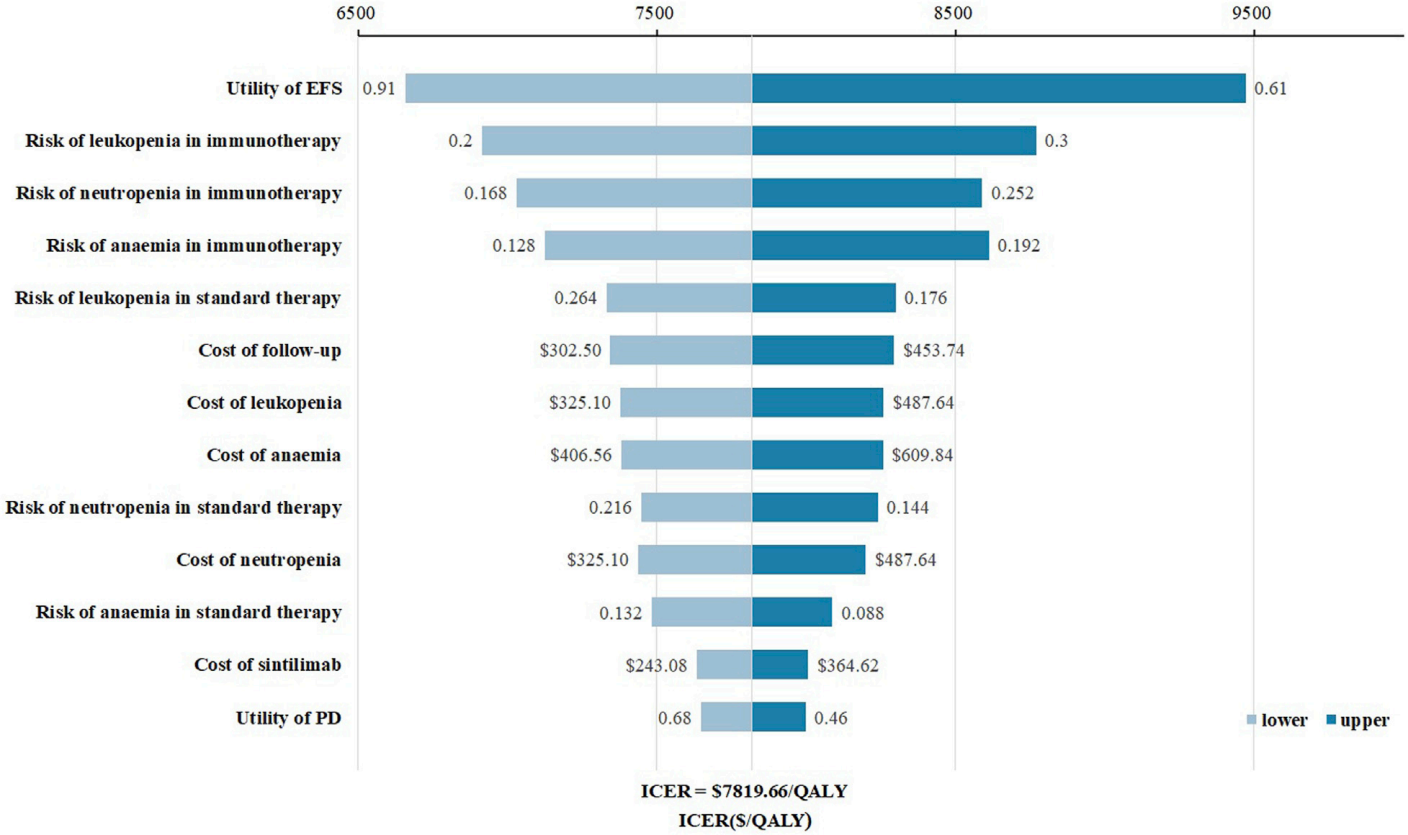
Tornado diagram for one-way sensitivity analysis. Abbreviations: EFS, event-free survival; PD, progressive disease.

**FIGURE 2:**
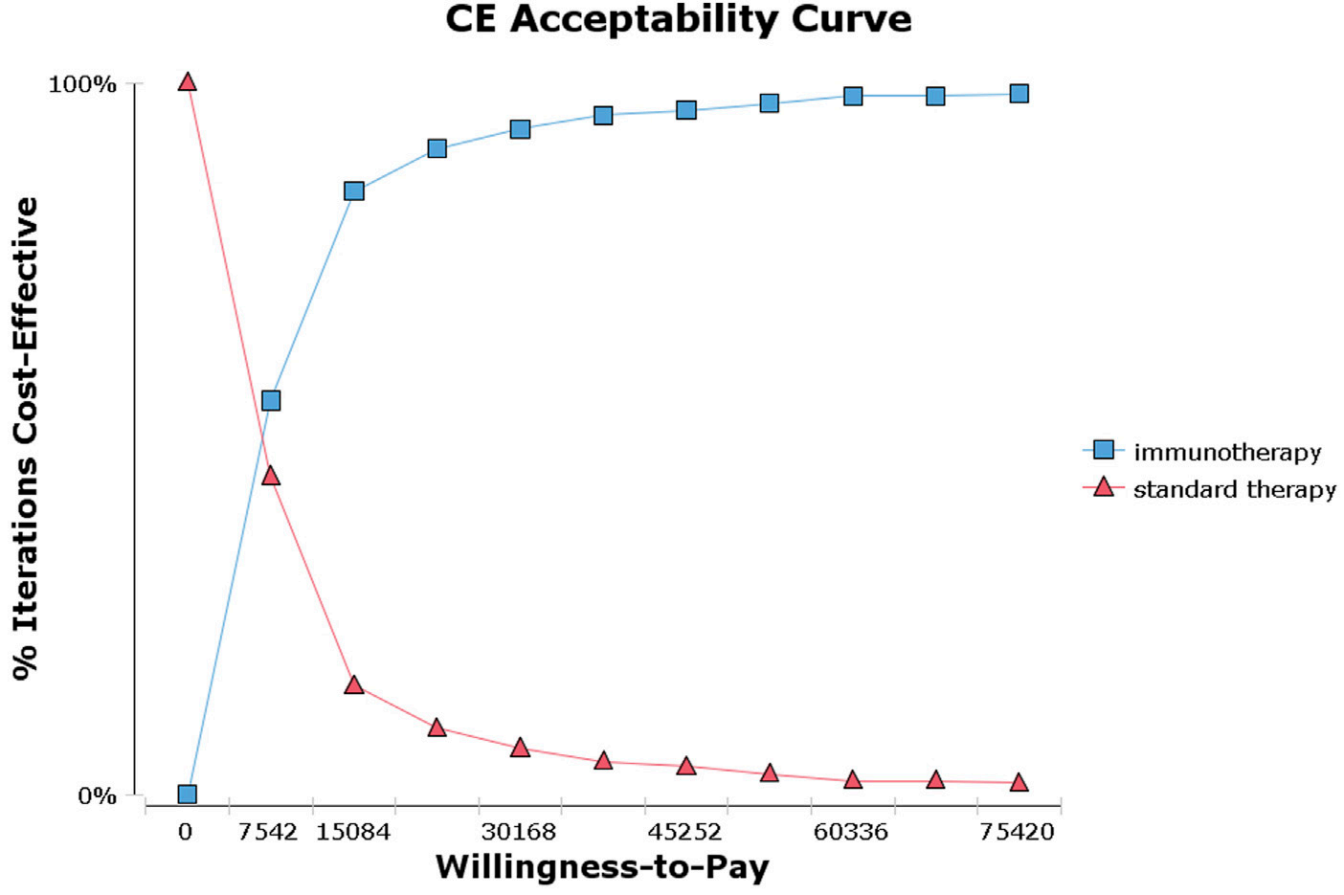
The results of Monte Carlo probabilistic sensitivity analysis.

**FIGURE 3:**
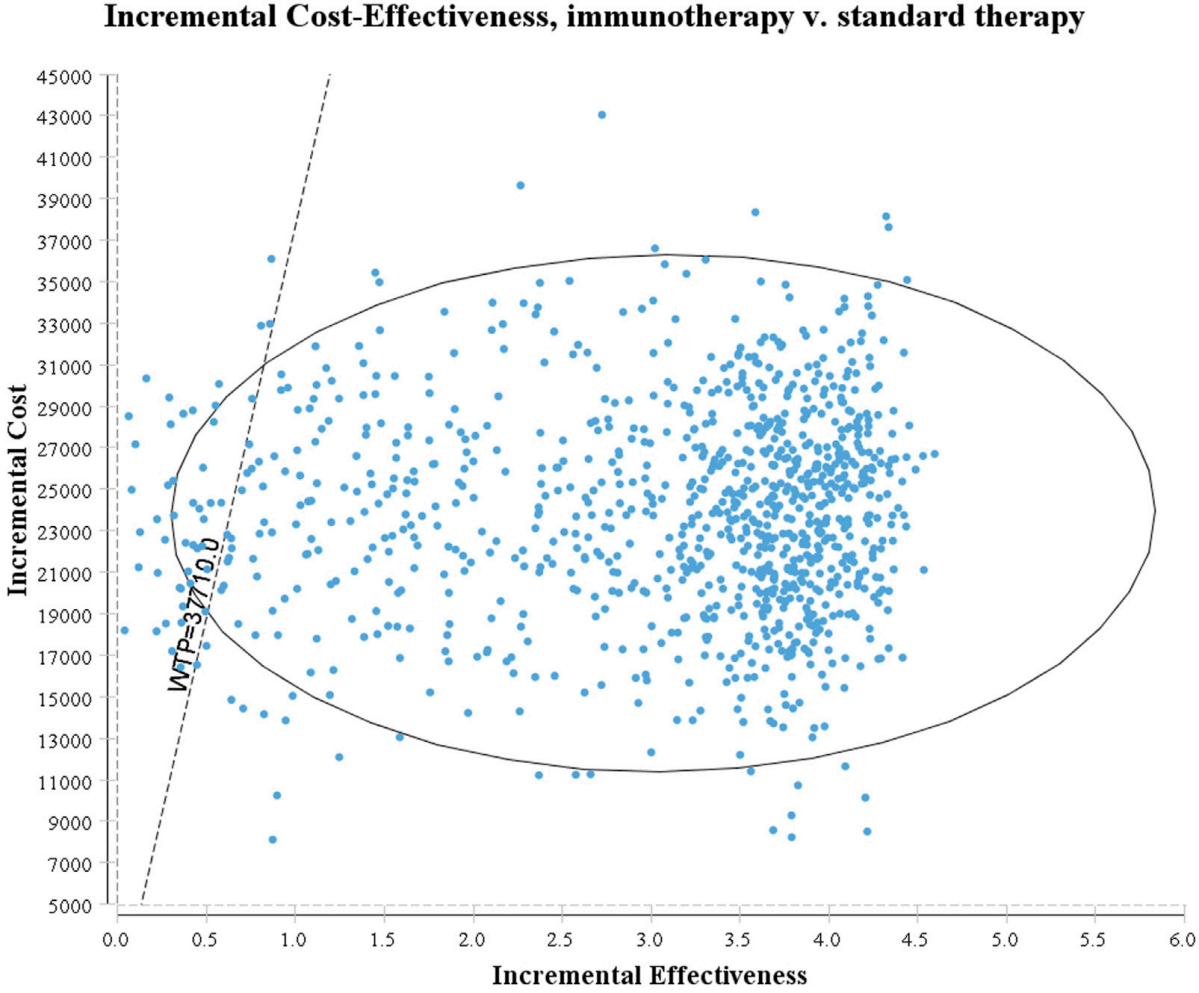
Acceptability curves for the choice of sintilimab or standard therapy strategies at different willingness-to-pay thresholds in patients with high-risk locoregionally advanced nasopharyngeal carcinoma.

The tornado diagram (Figure 1) presents the results of the one-way sensitivity analysis, highlighting the top three factors that had the most significant impact on cost-effectiveness: the utility of EFS, the risk of leukopenia in immunotherapy, and the risk of neutropenia in immunotherapy. As recommended by the World Health Organization (WHO) (Yang et al., 2020; Wu et al., 2020), the willingness-to-pay (WTP) threshold was set at three times China’s *per capita* GDP (equivalent to $37,710.00), derived from the Statistical Communiqué of the People’s Republic of China on the 2023 National Economic and Social Development (Gongsheng, 2025; National Bureau of Statistics, 2022). Based on this threshold, the addition of sintilimab demonstrated a 95.4% probability of being cost-effective by probabilistic sensitivity analysis (Figures 2, 3). In the subgroup analysis, consistent results were observed across different populations with varying PD-L1 CPS, TPS and EBV DNA levels (Supplementary Table S1).

## Discussion

In the past decade, immunotherapy has emerged as a pivotal breakthrough in cancer treatment (Bagchi et al., 2021). The CONTINUUM trial represents a significant milestone as the first phase 3 randomized trial to demonstrate the substantial clinical efficacy of a PD-1 inhibitor in the definitive treatment of patients with locoregionally advanced NPC (Liu et al., 2024). However, considering the economic burden associated with incorporating immunotherapy alongside chemotherapy, a comprehensive cost-effectiveness analysis is imperative. This analysis will help assess the balance between costs and potential benefits in terms of improved survival or enhanced quality of life, while also evaluating the general applicability of this approach within the healthcare landscape of China.

The present study represents the first known attempt to conduct an economic evaluation of sintilimab in combination with chemoradiotherapy as a treatment option for patients with locoregionally advanced NPC, from the perspective of the Chinese healthcare system. Using a Markov model, our baseline results indicated that the sintilimab regimen was more cost-effective compared to the standard regimen. The ICER amounted to $7,819.66 per QALY, which is significantly lower than the WTP threshold of $37710.00 per QALY in China. Notably, the ICER value was even below the one-time *per capita* gross domestic product (GDP), adding further significance to this finding, particularly in China. Furthermore, our one-way sensitivity analysis demonstrated that the cost of sintilimab had a minimal effect on the outcome, likely due to its relatively low cost. It is worth noting that even when all included parameters were varied within a given range, the sintilimab regimen remained consistently cost-effective, thus confirming the robustness of our model. Moreover, our probabilistic sensitivity analysis illustrated a high probability of 95.4% that the sintilimab group was cost-effective compared to the standard group. In summary, our findings strongly support that the first-line sintilimab with chemoradiotherapy is a cost-effective treatment strategy for Chinese patients with high-risk locoregionally advanced NPC.

Sintilimab for the treatment of NPC has not yet been included in China’s National Reimbursement Drug List. Given its clinical benefit and cost-effectiveness profile, this study provides economic evidence to support health policy decisions (e.g., reimbursement eligibility) for its use in locoregionally advanced NPC. Although drug pricing had a limited impact on the base results, one-way sensitivity analysis indicated that cost-effectiveness would further improve with price reduction. Considering the uneven economic development across regions different in China, including sintilimab in the medical insurance system and reducing its price could expand patient access and benefit a broader population.

Considering the new clinical evidence of first-line application of PD-1 inhibitor in locoregionally advanced NPC, as well as the unique geographic distribution of this disease, there is a lack of economic evaluations for this emerging protocol. As for recurrent or metastatic NPC, the combination of immunotherapy and chemotherapy has gradually become a standard first-line treatment in China following its approval by the CSCO guideline (Tang et al., 2021). Multiple studies have shown that these combined therapies are cost-effective options (Zhao et al., 2023; Zhu et al., 2022; Tang et al., 2023; Pei et al., 2023). Our study aligns with these findings and indicates that first-line PD-1 inhibitor in treatment of high-risk NPC would be cost-effective from the Chinese healthcare payer’s perspective.

Our one-way sensitivity analysis revealed that the utility of EFS, risks and costs of AEs, and cost of follow-up played significant roles in affecting the ICER. The occurrence of AEs is a critical factor affecting quality of life. Therefore, predicting and promptly managing the side effects could further enhance the cost-effectiveness of this first-line treatment. In the CONTINUUM trial, the use of sintilimab was associated with a higher incidence of grade 3 or higher AEs (74% in the immunotherapy group vs. 65% in the standard therapy group), which may explain the greater number of patients discontinuing concurrent chemoradiotherapy in the trial. This likely impacted the survival benefit reported for sintilimab and may also account for the more pronounced influence of AE risks on the ICER. The concurrent administration of multiple treatments might increase AE rates. Shifting the timing of immunotherapy to another treatment stage, such as adjuvant phase after sufficient recovery, might help minimize AEs and improve clinical outcomes, thereby potentially making immunotherapy more cost-effective. Additionally, in clinical practice, selecting personalized treatment plans and follow-up strategies tailored to patients’ clinical conditions and financial status may help reduce their economic burden.

Recently, the DIPPER trial results presented at the 2024 annual ASCO meeting provided promising findings (Jun et al., 2024). Adjuvant camrelizumab was found to significantly prolong EFS compared to chemoradiotherapy alone as a first-line treatment for locoregionally advanced NPC. Despite the high survival benefits associated with immunotherapy combinations, there is a substantial financial burden on both patients and the healthcare system. The latest data from the National Bureau of Statistic of China in 2022 indicates that annual healthcare costs have risen over 8 trillion yuan ($1 trillion) (China Statistical Yearbook, 2022). Moreover, as more clinical trial results are released, selecting the most suitable first-line immunotherapy regimen for locoregionally advanced NPC becomes increasingly challenging. Conducting an economic evaluation within the context of close clinical efficacy becomes vital. Studies have shown that toripalimab, camrelizumab or tislelizumab in combination with chemotherapy all offer significant survival improvement for recurrent or metastatic NPC (Mai et al., 2023; Yang et al., 2021; Yang et al., 2023). Han et al. revealed that, among three chemo-immunotherapy groups, toripalimab regimen was the most cost-effective option, followed by camrelizumab and tislelizumab groups (Han et al., 2023). This information aids patients, clinicians, and policymakers in making informed decisions. We will update our analysis once the detailed data from the DIPPER trial are published. This will provide additional evidence of the pharmacoeconomics for incorporating immunotherapy into locoregionally advanced NPC treatment, thereby facilitating optimal clinical practices.

According to the published research, PD-L1 expression level have been identified as a potential biomarker in NPC (Chen Y-P. et al., 2019; Mai et al., 2023; Hsu et al., 2017). In light of this, we conducted subgroup analyses based on PD-L1 expression using data from the CONTINUUM trial. Our analyses showed that the sintilimab group was a cost-effective choice across all PD-L1 expression populations, including those with PD-L1 CPS or TPS <1. It is noteworthy that the ICER value in PD-L1 TPS ≥20 population was lower than those in other populations, suggesting that this specific group had the highest level of cost-effectiveness. Furthermore, sintilimab was cost-effective in the PD-L1 unknown status population, which may be attributed to the generally high-level PD-L1 expression characteristic of NPC (Supplementary Table S2). Additionally, plasma EBV-DNA levels also served as a prognostic biomarker. The CONTINUUM trial revealed that patients with lower baseline EBV-DNA level derived greater clinical benefits (Chen Y-P. et al., 2019; Yang et al., 2023). We performed subgroup analyses to confirm these findings, revealing that patients with lower baseline levels had a higher probability of cost-effectiveness (Supplementary Table S2). However, aside from baseline, the expression level after induction chemotherapy or radiotherapy also play a role in prognosis. Continued research is necessary to investigate other relevant factors and their impact on prognosis and cost-effectiveness.

We recognize that there are still limitations in current study, despite our best efforts to consider the impacts of multiple factors. Firstly, the survival benefits observed in the CONTINUUM trial were obtained with a median follow-up time of 41.9 months. In our model, we extended the follow-up time using the available data for the entire treatment cycle. However, it is possible that the actual survival data may differ. To mitigate this potential bias, we employed five different distributions to fit the survival curves and selected the best-fitting distribution. In addition, although GetData Graph Digitizer is a common method to extract survival data from Kaplan-Meier curves, it carries potential limitations such as digitization errors and insufficient data point density. To address these issues, we implemented corresponding solutions, including repeated extractions with averaging, high-resolution original images, and calibration against survival rates reported in the original publication. However, as with most cost-effectiveness analysis studies (Wu et al., 2018; Wan et al., 2019a; Wan et al., 2019b; Sarfaty et al., 2018), this potential error is unavoidable. With the development of cost-effectiveness analysis, we believe that this issue will be resolved. Secondly, the CONTINUUM trial was conducted in endemic regions, and our analysis was also developed within the same area. Therefore, these results should be cautiously applied to regions with differing demographics and healthcare systems. Thirdly, immune-related AEs could be financially burdensome but occur at a relatively low incidence. We excluded immune-related AEs with an incidence of less than 3%, as their impact on the overall outcomes would likely be minimal due to the low occurrence rate. Fourthly, in subgroup analysis, we made an assumption that all other elements were similar among the subgroups, except for the hazard ratio for EFS. However, it is crucial to recognize that this subgroup analysis is exploratory in nature. Furthermore, the exploratory subgroup analyses tend to have small sample sizes and therefore should be interpreted with caution. To address this limitation, we will update analyses with emerging subgroup data and validate findings in larger cohorts in future studies. Fifth, we acknowledge that incorporating comparisons with other PD-1 inhibitors (such as camrelizumab) would enhance the clinical relevance of our findings. However, no head-to-head trial data are currently available to support such comparisons in the NPC setting. Our analyses will be updated as new evidence emerges from future studies.

In conclusion, induction-concurrent chemoradiotherapy with sintilimab for high-risk locoregionally advanced NPC represents a cost-effective choice.

## Data Availability

The original contributions presented in the study are included in the article/Supplementary Material, further inquiries can be directed to the corresponding author.
