# Supplementary material for: Cost-effectiveness analysis of sintilimab additional to chemoradiotherapy in high-risk locoregionally advanced nasopharyngeal carcinoma
Source: Front Pharmacol. 2025 Jul 9;16:1548710. doi: 10.3389/fphar.2025.1548710 (PMC12283321; doi:10.3389/fphar.2025.1548710)
Supplement: Supplementary file 1 [file Supplementaryfile1.zip › Supporting Table 1.docx]

**Supporting Table 1.** Summary of statistical goodness-of-fit of K-M curve in CONTINUUM.

|  | **Exponential** | **Weibull** | **Log-logistic** | **Lognormal** | **Gompertz** |
| --- | --- | --- | --- | --- | --- |
| **Sintilimab VS Standard therapy** | | | | | |
| **Sintilimab OS curve** | | | | | |
| AIC | -576.3301 | -591.9314 | -597.2082 | -593.9497 | -593.733 |
| BIC | -571.5912 | -584.8231 | -590.0998 | -586.8413 | -586.6247 |
| **Standard therapy OS curve** | | | | | |
| AIC | -360.3266 | -549.318 | -552.2933 | -539.1077 | -409.5604 |
| BIC | -355.5877 | -542.2096 | -545.185 | -531.9994 | -402.4521 |
| **Sintilimab EFS curve** | | | | | |
| AIC | -427.8627 | -517.4534 | -558.1817 | -527.1646 | -363.5895 |
| BIC | -423.1238 | -510.3451 | -551.0733 | -520.0562 | -356.4812 |
| **Standard therapy EFS curve** | | | | | |
| AIC | -362.4824 | -367.2734 | -400.4939 | -379.2661 | -384.6076 |
| BIC | -357.769 | -360.2033 | -393.4237 | -372.196 | -377.5375 |

Abbreviation: AIC, Akaike’s information criterion; BIC, Bayesian information criterion; EFS, Event-free survival; OS, overall survival.
