# Supplementary material for: Cost-effectiveness analysis of sintilimab additional to chemoradiotherapy in high-risk locoregionally advanced nasopharyngeal carcinoma
Source: Front Pharmacol. 2025 Jul 9;16:1548710. doi: 10.3389/fphar.2025.1548710 (PMC12283321; doi:10.3389/fphar.2025.1548710)
Supplement: Supplementary file 1 [file Supplementaryfile1.zip › Supporting Table 2.docx]

**Supporting Table 2. Subgroup analyses results in different PD-L1 CPS, TPS and EBV DNA level.**

| **Subgroup** | **No. of patients with event/total** | | **EFS HR (95% CI)** | **ICER per QALY (95% CI)** | **Cost-effectiveness probability at WTP $37710/QALY** | |
| --- | --- | --- | --- | --- | --- | --- |
|  | **Sintilimab**  **(33/210)** | **Standard therapy**  **(53/215)** |  |  |  |  |
| **CPS** |  |  |  |  | |  |
| <1 | 7/26 | 8/30 | 1.04 (0.38-2.88) | 6058.97 (5358.20-7611.90) | | 95.1% |
| 1-19 | 10/72 | 16/61 | 0.52 (0.23-1.14) | 5515.80 (5183.10-6156.76) | | 97.5% |
| ≥20 | 2/32 | 4/36 | 0.53 (0.10-2.87) | 5526.86 (5025.62-7604.60) | | 97.4% |
| Unknown | 14/80 | 25/88 | 0.59 (0.31-1.13) | 5592.64 (5277.33-6147.07) | | 96.3% |
| **CPS** |  |  |  |  | |  |
| <1 | 7/26 | 8/30 | 1.04 (0.38-2.88) | 6058.97 (5358.20-7611.90) | | 95.1% |
| ≥1 | 12/104 | 20/97 | 0.54 (0.26-1.11) | 5537.88 (5218.67-6127.63) | | 97.0% |
| Unknown | 14/80 | 25/88 | 0.59 (0.31-1.13) | 5592.64 (5277.33-6147.07) | | 96.3% |
| **CPS** |  |  |  |  | |  |
| <10 | 14/73 | 21/73 | 0.64 (0.33-1.27) | 5646.76 (5300.58-6281.07) | | 96.1% |
| ≥10 | 5/57 | 7/54 | 0.66 (0.21-2.09) | 5668.24 (5159.23-7001.47) | | 96.0% |
| Unknown | 14/80 | 25/88 | 0.59 (0.31-1.13) | 5592.64 (5277.33-6147.07) | | 96.3% |
| **TPS** |  |  |  |  | |  |
| <1 | 9/42 | 11/45 | 0.85 (0.35-2.05) | 5867.57 (5323.72-6968.58) | | 95.8% |
| 1-19 | 9/61 | 13/51 | 0.59 (0.25-1.37) | 5592.64 (5206.84-6374.61) | | 96.3% |
| ≥20 | 1/27 | 4/31 | 0.26 (0.03-2.37) | 5218.67 (4938.45-7226.03) | | 98.6% |
| Unknown | 14/80 | 25/88 | 0.59 (0.31-1.13) | 5592.64 (5277.33-6147.07) | | 96.3% |
| **TPS** |  |  |  |  | |  |
| <1 | 9/42 | 11/45 | 0.85 (0.35-2.05) | 5867.57 (5323.72-6968.58) | | 95.7% |
| ≥1 | 10/88 | 17/82 | 0.54 (0.25-1.18) | 5537.88 (5206.84-6195.35) | | 97.0% |
| Unknown | 14/80 | 25/88 | 0.59 (0.31-1.13) | 5592.64 (5277.33-6147.07) | | 96.3% |
| **TPS** |  |  |  |  | |  |
| <10 | 16/85 | 21/76 | 0.67 (0.35-1.28) | 5678.94 (5323.72-6290.50) | | 95.9% |
| ≥10 | 3/45 | 7/51 | 0.46 (0.12-1.80) | 5448.92 (5050.22-6758.16) | | 97.9% |
| Unknown | 14/80 | 25/88 | 0.59 (0.31-1.13) | 5592.64 (5277.33-6147.07) | | 96.3% |
| **Baseline EBV DNA** |  |  |  |  | |  |
| <4000 | 11/132 | 32/144 | 0.34 (0.17-0.68) | 5312.17 (5111.11-5689.62) | | 98.1% |
| ≥4000 | 18/64 | 1758 | 0.99 (0.51-1.92) | 6009.33 (5504.72-6860.23) | | 95.6% |
| Unknown | 4/14 | 4/13 | 0.98 (0.24-3.92) | 5999.34 (5194.99-8312.63) | | 95.6% |

Abbreviation: CI, confidence interval; CPS, combined positive score; EBV, Epstein-Barr virus; EFS HR, event-free survival hazard ratio; ICER, incremental cost-effectiveness ratio; QALY, quality-adjusted life-year; TPS, tumor proportion score; WTP, Willingness-to-pay.
