# Supplementary figures and images for: Cost-effectiveness analysis of sintilimab additional to chemoradiotherapy in high-risk locoregionally advanced nasopharyngeal carcinoma
Source: Front Pharmacol. 2025 Jul 9;16:1548710. doi: 10.3389/fphar.2025.1548710 (PMC12283321; doi:10.3389/fphar.2025.1548710)

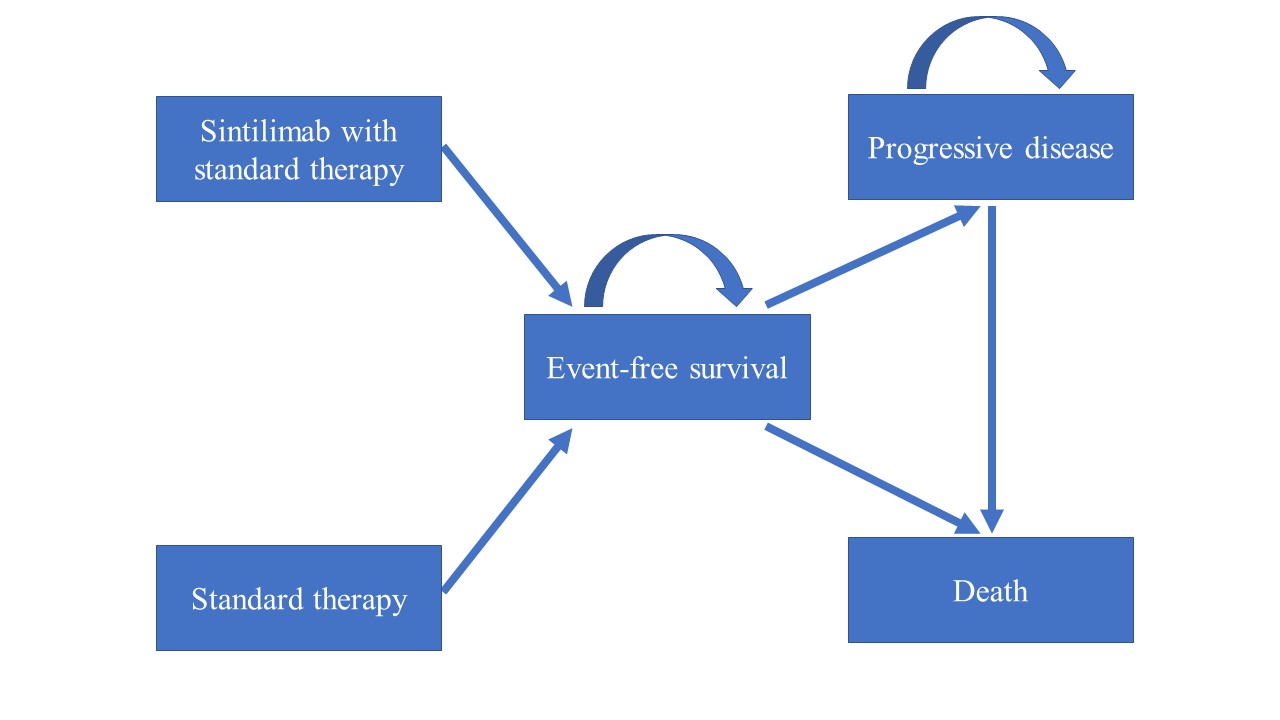

Supplement: Supplementary file 1 [file Supplementaryfile1.zip › Supporting Figure 1.jpg]
